# Plasma nontargeted metabolomics study of H1N1 and H3N2 influenza in children

**DOI:** 10.3389/fcimb.2025.1537726

**Published:** 2025-04-04

**Authors:** Yaping Li, Jiaxin Li, Ting Li, Chenrui Liu, Jiayi Du, Yuxin Li, Yuan Chen, Yufeng Zhang, Xiaoyan Wang, Xinyu Wang, Xiaoli Jia, Huiling Deng

**Affiliations:** ^1^ Department of Infectious Diseases, Xi’an Jiaotong University Second Affiliated Hospital, Xi’an, China; ^2^ Department of Infectious Diseases, Xi’an Children’s Hospital, Xi’an, China; ^3^ School of Public Health, Shaanxi University of Chinese Medicine, Xi’an, China; ^4^ Epidemiology of Microbial Disease, Yale University School of Public Health, New Haven, CT, United States; ^5^ Department of Neurology, Xi’an Children’s Hospital, Xi’an, China; ^6^ Department of Pediatrics, Xi’an Central Hospital, Xi’an, China

**Keywords:** influenza virus, plasma metabolomics, differentially abundant metabolites, biomarker, early diagnosis

## Abstract

**Background:**

This study used a nontargeted metabolomic approach to investigate small molecular metabolites in the peripheral blood of pediatric patients with influenza. By comparing these metabolites with those in healthy children, potential biomarkers for the early detection and diagnosis of influenza were explored.

**Methods:**

Plasma samples were collected from 47 children with H1N1 influenza, 40 with H3N2 influenza, and 40 healthy controls at Xi’an Children’s Hospital, Xi’an Jiaotong University Second Affiliated Hospital, and Xi’an Central Hospital between May and September 2023. Nontargeted metabolomic detection and analysis were performed.

**Results:**

In the H1N1 group, 14 glycerophospholipid metabolites were significantly altered compared to controls, with 11 (78.5%) markedly downregulated. These downregulated metabolites showed negative correlations with inflammatory markers, including white blood cell (WBC) count, neutrophils, C-reactive protein (CRP), and Procalcitonin (PCT), whereas the upregulated metabolite PC(P-18:1(9Z)/16:0) showed positive correlations with validation markers. In the H3N2 group, 12 glycerophospholipid metabolites were significantly altered, with 9 being downregulated. The downregulated LysoPC(20:0/0:0) showed a positive correlation with alanine aminotransferase (ALT) but a negative correlation with WBC count, while the upregulated metabolite LysoPA(18:1(9Z)0:0) correlated positively with ALT, aspartate aminotransferase (AST), and lactate dehydrogenase (LDH).

**Conclusions:**

Distinct metabolomic profiles were identified in pediatric H1N1 and H3N2 influenza cases compared to healthy controls. Specific glycerophospholipid metabolites were closely associated with inflammatory and liver function markers, highlighting their potential as biomarkers for disease monitoring and early diagnosis.

## 1 Introduction

Influenza is an acute respiratory infectious disease caused by the influenza virus that is highly pathogenic and has a high mortality rate. As one of the major global public health threats, influenza viruses are widely distributed worldwide. In particular, influenza A and B viruses are the main causes of human respiratory infectious diseases (Jansen et al., 2019). During the period from week 27 in 1996 to week 26 in 2021, 39,637,339 influenza samples were monitored worldwide (Chen C. et al., 2023). Each year, 5–10% of adults and 20–30% of children were infected, and 290,000–650,000 people died (Chen et al., 2022). Among influenza A strains, the H1, H2 and H3 subtypes are the main pathogens causing human influenza (Cui et al., 2016). Currently, most cases of influenza in humans are caused by the avian influenza viruses H1N1 and H3N2 (Flerlage et al., 2021). The severity and mortality of influenza A virus in infants and older individuals are significantly greater than in young and middle-aged people, who have relatively mild symptoms (Worobey et al., 2014). In 1968, a new subtype of influenza A virus, H3N2, emerged, causing a worldwide pandemic and >1 million fatalities (Alymova et al., 2016). Recent epidemiological data from China further highlight the seasonal burden of H1N1 and H3N2 infections. According to the latest influenza surveillance report from the Chinese Center for Disease Control and Prevention (China CDC), as of December 29, 2024, the influenza virus positivity rate in both northern and southern regions has been increasing, with A(H1N1) pdm09 as the predominant circulating subtype. A total of 171 influenza-like illness (ILI) outbreaks have been reported nationwide. During the period from April 1 to December 29, 2024, 97.0% of A(H1N1) pdm09 strains were found to be genetically similar to A/Victoria/4897/2022, while 56.0% of A(H3N2) strains were similar to A/Thailand/8/2022 (egg-propagated strain) (China CDC Weekly, 2024).

Although the specific pathogenesis of influenza remains unclear, it has been shown that the main mechanism is that the virus invades respiratory epithelial cells to cause lung inflammation and damage, while the body’s corresponding immune response also affects lung inflammation (Kalil and Thomas, 2019). During the progression of influenza, various small molecules in the host may play important immunoregulatory roles. Children’s innate immune recognition receptors, such as Toll-like receptor 3 and 7, can influence the development of signal transduction pathways, reducing the body’s ability to clear influenza virus, induce an excessive inflammatory response, and increase the risk of secondary infection (Coates et al., 2015). CD4^+^ T cells lacking arginase (Arg)1 proliferate faster during the T helper (Th)1 life cycle, produce the corresponding Th1 cytokines earlier, and enter the contraction phase more rapidly after influenza virus infection. Accelerated Th1-induced transformation can control the virus to some extent, enhance the production of interleukin-10, and prevent excessive tissue damage (West et al., 2023). Therefore, it is necessary to study the regulatory effects of various small molecule substances in the body.

As an emerging technology, metabolomics has played an increasingly important role in revealing the metabolic response of the body under physiological and pathological conditions. This approach involves the measurement of small molecule chemical substances, including endogenous and exogenous compounds. Endogenous compounds are usually produced by endogenous synthesis or degradation and are critical for the growth and development of the body and participate in important physiological functions (Wishart, 2019). In addition, metabolomics studies do not need sample types and are suitable for studies of most samples (Liu and Locasale, 2017). To date, metabolome research techniques include chemical analysis of metabolites and data analysis. The main techniques used are divided into two parts: nuclear magnetic resonance and mass spectrometry (MS), liquid chromatography (LC), gas chromatography, or capillary electrophoresis (Miggiels et al., 2019). Currently, metabolomics is applied to evaluate the therapeutic effect of drugs on influenza virus infection (Beale et al., 2019; Feng et al., 2023) and to explore abnormal metabolic pathways after influenza virus infection through cell and animal experiments (Karimi et al., 2022).

Lipid metabolism plays a crucial role in influenza virus infection and replication. As an enveloped RNA virus, influenza virus heavily relies on host lipid metabolic pathways to complete its life cycle. Lipids are essential for viral entry, membrane fusion, replication, and virion assembly, making lipidomic alterations a key factor in disease pathogenesis. Recent studies have demonstrated that host lipid metabolism, particularly glycerophospholipid metabolism, is significantly altered during influenza infection, affecting both immune response modulation and viral propagation (Chen X. Y. et al., 2023; Kawabata et al., 2023). Several metabolic pathways, including fatty acid metabolism, cholesterol biosynthesis, and phospholipid remodeling, have been identified as potential targets for influenza pathogenesis research (Zhou et al., 2021).

Given the critical involvement of lipid metabolism in influenza progression, this study applies untargeted metabolomics analysis to investigate plasma metabolic alterations in peripheral blood samples of Chinese Han children after influenza virus infection. The identification of biomarkers may contribute to early diagnosis, predicting prognosis, disease risk, disease progression and treatment outcomes, and providing new insights into the continuous pathophysiological process of viral pneumonia.

## 2 Methods

### 2.1 Study subjects and sample collection

Peripheral blood samples were collected from 47 children with influenza A (H1N1) and 40 children with influenza A (H3N2) who were hospitalized at Xi’an Children’s Hospital, Xi’an Jiaotong University Second Affiliated Hospital, and Xi’an Central Hospital between May and September 2023, Additionally, 40 healthy control children were included during the same period. Influenza cases were diagnosed according to the *Expert Consensus on the Diagnosis and Treatment of Children Influenza (2020 Edition)* and were further classified into mild and severe groups based on clinical severity (Zhou et al., 2021).

Mild cases exhibited influenza-like symptoms with systemic manifestations more pronounced than the common cold but without complications. Severe cases were defined as those meeting at least one of the following criteria: (1) persistent high fever lasting more than three days, accompanied by severe cough, purulent or bloody sputum, or chest pain; (2) significant respiratory distress, including markedly increased respiratory rate, dyspnea, or cyanosis; (3) neurological symptoms such as altered mental status, lethargy, irritability, or convulsions; (4) severe gastrointestinal symptoms, including frequent vomiting, diarrhea, or signs of dehydration; (5) complications such as pneumonia or encephalitis; or (6) exacerbation of pre-existing underlying medical conditions (National Clinical Medical Research Centre for Respiratory Diseases, Respiratory Group of the Paediatrics Branch of the Chinese Medical Association, 2020).

Throat swab samples were collected from all children in the case group after admission to hospital. The throat swab samples were tested for influenza virus antigen or viral RNA by the hospital laboratory department or the Centers for Disease Control and Prevention. The families of all the children tested were informed of the purpose of the study and provided written informed consent. This study was approved by the Medical Ethics Committee of Xi’an Children’s Hospital, Xi’an Jiaotong University Second Affiliated Hospital and Xi’an Central Hospital.

### 2.2 Clinical data collection and analysis

All clinical data were obtained from the original medical records of patients and relevant laboratory test results after admission. Clinical physicians obtained the medical records through face-to-face interviews with the patients or their family members, and relevant laboratory test results were obtained from the patients’ blood samples. SPSS 23.0 software was used for statistical analysis, and the distribution characteristics of influenza were analyzed using descriptive epidemiological methods. Measurement data were expressed as mean ± standard deviation (SD) or median (Q25, Q75) and were compared by *t-test* (normally distributed data) and Mann–Whitney *U* test (non-normally distributed data). The χ^2^ test was used for numerical data, α=0.05.

### 2.3 Collection and preparation of plasma samples

Blood samples (2 mL/tube) were collected in EDTA anticoagulant tubes under fasting conditions in the morning. After gentle mixing, the samples were centrifuged at 4°C, and the supernatant was transferred into enzyme-free EP tubes. All plasma samples were stored in a -80°C medical freezer.

### 2.4 Metabolite chemical analysis and data analysis

Test solutions were prepared by mixing 100 µL of plasma with 400 µL of methanol/acetonitrile (1:1) extraction solution containing isotope-labeled internal standards, followed by vortexing and centrifugation at 3500 RPM for 10 minutes at 4°C. The supernatant was collected for further analysis. QC samples were prepared by pooling equal volumes of supernatant from all samples. Target compound separation was conducted using a Waters ACQUITY UPLC BEH Amide column (Thermo Fisher Scientific), and mass spectrometry was performed with an Orbitrap Exploris 120 system (Thermo), with primary and secondary MS data acquired using Xcalibur software (version 4.4).

Process QC ensured minimal differences in internal standard peaks between QC samples and blank samples. Data QC involved analyzing the PCA scores, PCA-X distribution, and correlation of QC samples. Data quality was confirmed by QC variation within ±2 SD and correlation coefficients close to 1.

Raw data were converted into mzXML format using ProteoWizard and analyzed using an in-house R package for peak extraction, alignment, and integration. Metabolites were identified through KEGG and MetaboAnalyst databases and screened using OPLS-DA (VIP>1.000, P<0.05). Metabolic pathway enrichment was performed using MetaboAnalyst (https://www.metaboanalyst.ca/). Cytoscape software (version 3.7.1) was used to visualize differences in metabolite expression between groups.

### 2.5 Correlation analysis of metabolites and clinical indicators

We conducted Pearson correlation tests to analyze the relationship between the most prevalent type of differential metabolites (glycerophospholipids in this study) and clinical test indicators [including blood routine, liver function, C-reactive protein (CRP), procalcitonin (PCT), etc.] in patients sampled from the H1N1 group versus normal control group and the H3N2 group versus normal control group. P<0.05 was considered statistically significant.

## 3 Results

### 3.1 Clinical characteristics of H1N1 and H3N2 influenza patients

This study included 47 H1N1 influenza patients, 40 H3N2 influenza patients, and 40 healthy controls, with cases divided into severe and mild groups. Among H1N1 cases, there were 10 severe and 37 mild, while the H3N2 group comprised 11 severe and 29 mild cases. Blood samples were collected for untargeted metabolomic analysis.

Forty-seven H1N1 cases were included (61.7% male). Ages ranged from 3 months to 14 years (median: 3.83 years). Patients predominantly lived in urban areas (72.3%) and exhibited symptoms such as fever (100%), cough (83%), and vomiting (40.4%). Fever duration ranged from 1 to 8 days, with an average peak temperature of 39.44°C. Immune profiling revealed significantly lower white blood cell (WBC) and neutrophil counts, with elevated monocyte percentages compared to controls (*P*<0.01) (**Supplementary Table 1**). Forty H3N2 cases were analyzed (57.5% male), with an age range of 5 months to 12 years (average: 4.66 years). Symptoms mirrored those of H1N1, with fever (100%) and cough (82.5%) being most prevalent. Fever duration ranged from 1 to 15 days, with an average peak temperature of 39.6°C in **Table 1**. Immune parameters showed reduced WBC, neutrophil, and lymphocyte counts, with elevated monocyte percentages compared to controls (*P*<0.01) (**Supplementary Table 1**). However, no significant statistical differences were observed in immune cell counts between the H1N1 and H3N2 groups (P>0.05).

**Table 1:** Clinical information for the H1N1 and H3N2 patients.

Residence type	H1N1 group	H3N2 group	Control group	*P1* value	*P2* value	*P3* value
					
Nursery	10 (21.3%)	7 (17.5%)	–	0.409	–	–
Diaspora	18 (38.3%)	21 (52.5%)	–			
Student	19 (40.4%)	12 (30%)	–			
Residence				0.810	–	–
Towns	34 (72.3%)	28 (70%)	–			
Rural or township	13 (27.7%)	12 (30%)	–			
Gender				0.690	0.527	0.822
male, male	29 (61.7%)	23 (57.5%)	22 (55%)			
women, women	18 (38.3%)	17 (42.5%)	18 (45%)			
Age, years	3.83 (2.25,6.00)	4.17 (2.38,6.77)	5 (3.25,6.00)	0.624	0.138	0.472
Thermal history, day	3 (2,5)	3 (2,5)	–	0.398	–	–
Highest temperature	39.44 ± 0.59°C*	39.36 ± 0.67°C*	–	0.576	–	–
Fever	47 (100%)	40 (100%)	–			
Cough	39 (83%)	33 (82.5%)	–	0.953	–	–
Twitching	7 (14.9%)	9 (22.5%)	–	0.361	–	–
Sore throat	1 (2.1%)	3 (7.5%)	–	0.233	–	–
Muscle soreness	2 (4.3%)	1 (2.5%)	–	0.655	–	–
Vomiting	19 (40.4%)	15 (37.5%)	–	0.780	–	–
Abdominal pain	4 (8.5%)	5 (12.5%)	–	0.543	–	–
Diarrhea	3 (6.4%)	1 (2.5%)	–	0.389	–	–
dizziness	3 (6.4%)	4 (10%)	–	0.536	–	–
Headache	4 (8.5%)	2 (5%)	–	0.467	–	–
Highly sensitive C-reactive protein	1.7 (<0.5, 8.21)	5.25 (0.70,11.07)	–	0.279	–	–
ALT	16 (14.75, 21)	15 (12,22)	11.20 ± 3.31	0.240	0.000	0.000
AST	41 (33, 55)	39.50 (30.0, 50.50)	23.20 ± 4.22	0.421	0.000	0.000
CK	97.0 (59.75, 168.25)	101.00 (76.25, 142.50)	80.70 ± 31.12	0.649	0.031	0.002
CK-MB	21.0 (16.0, 27.0)	17.50 (15.0, 23.0)	14.08 ± 3.98	0.059	0.000	0.000
LDH	268.0 (237.0, 304.0)	240.50 (212.50, 284.25)	219.50 (206.50,242.00)	0.032	0.000	0.033
Creatinine	34.43 ± 8.69*	34.80 ± 10.26*	32.68 ± 7.26	0.859	0.316	0.289
Uric acid	254 (216.0, 302.50)	258 (209.25, 296.25)	–	0.736	–	–
PCT	0.15 (0.08, 0.27)	0.12 (0.05, 0.25)	–	0.339	–	–
Erythrocyte sedimentation rate	11 (7, 14)	11 (6,15)	–	0.770	–	–

*Normally distributed data. *P1* value, H1N1 patients compared with H3N2 patients; *P2* value, H1N1 patients compared with control patients; *P3* value, H3N2 patients compared with control patients.

In comparison, the control group (40 healthy individuals) had an age range of 2 months to 8 years, with a gender distribution of 55% male and 45% female. There were no significant differences in age and gender between the case groups and the control group. For liver function clinical indicators, a comparison between the influenza groups and the control group showed that ALT, AST, CK, CK-MB, and LDH levels were significantly different between the H1N1 group and the control group, and the H3N2 group and the control group (P<0.05).

### 3.2 Glycerophospholipid metabolism pathway in H1N1 patients

**Figure 1:**
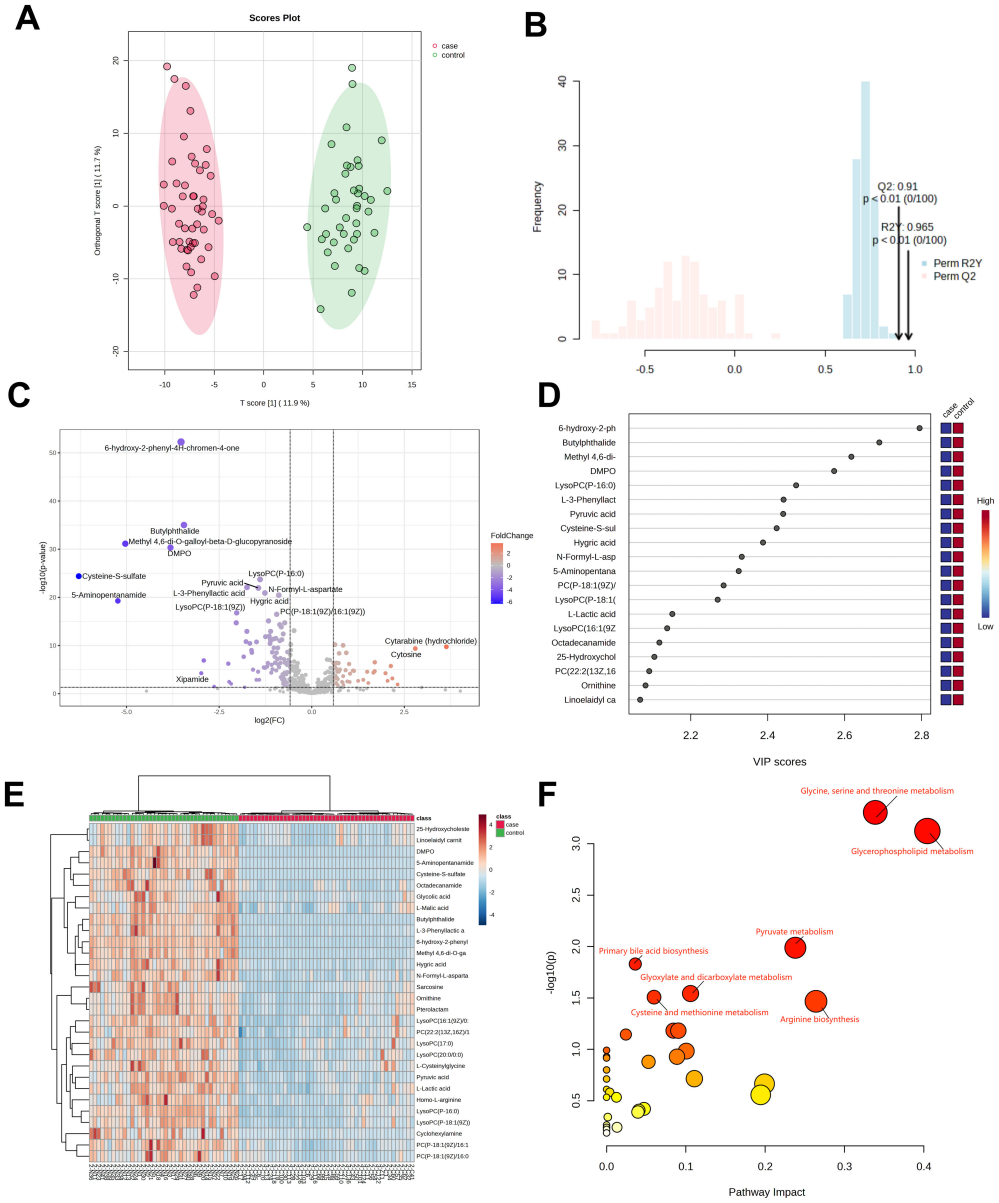
Metabolomic analysis of patients in the H1N1 group. OPLS-DA score diagram of pediatric H1N1 influenza patients and healthy controls **(A, B)**;Volcano plot of differential metabolites between children with H1N1 influenza and the healthy control group **(C)**; Top 20 metabolites with the highest VIP values **(D)**; Heatmap of the top 30 different metabolites between children with H1N1 influenza and healthy controls **(E)**; Enrichment analysis of the differentially abundant metabolite pathways in the H1N1 group vs. healthy controls **(F)**.

Plasma samples from 47 H1N1 patients and 40 healthy controls underwent untargeted metabolomics analysis. A total of 563 metabolites were detected and analyzed using orthogonal PLS-DA (R²Y=0.965, Q²=0.91; *P*<0.01), as shown in **Figures 1A, B**, demonstrating clear separation between the H1N1 and control groups. Differentially abundant metabolites (n=81) were identified based on criteria of VIP>1, P<0.05, fold change >1.5 or <2/3, and M2 score >0.7. Among these, 27 metabolites were upregulated, and 54 were downregulated (**Figure 1C**).

Of the 81 differentially abundant metabolites, 14 (17.2%) were glycerophospholipids, with 78.5% of them significantly downregulated in H1N1 patients. The top 20 metabolites with the highest VIP scores in the OPLS-DA model are depicted in **Figure 1D**, while the top 30 differential metabolites are visualized as a heatmap in **Figure 1E**.

KEGG pathway analysis revealed that the differentially abundant metabolites were primarily enriched in glycerophospholipid metabolism, pyruvate metabolism, glycine, serine, and threonine metabolism, and primary bile acid biosynthesis (**Figure 1F**). These findings strongly suggest that H1N1 influenza disrupts the glycerophospholipid metabolism pathway, potentially contributing to the disease’s pathophysiology.

### 3.3 Glycerophospholipid metabolism pathway in H3N2 patients

**Figure 2:**
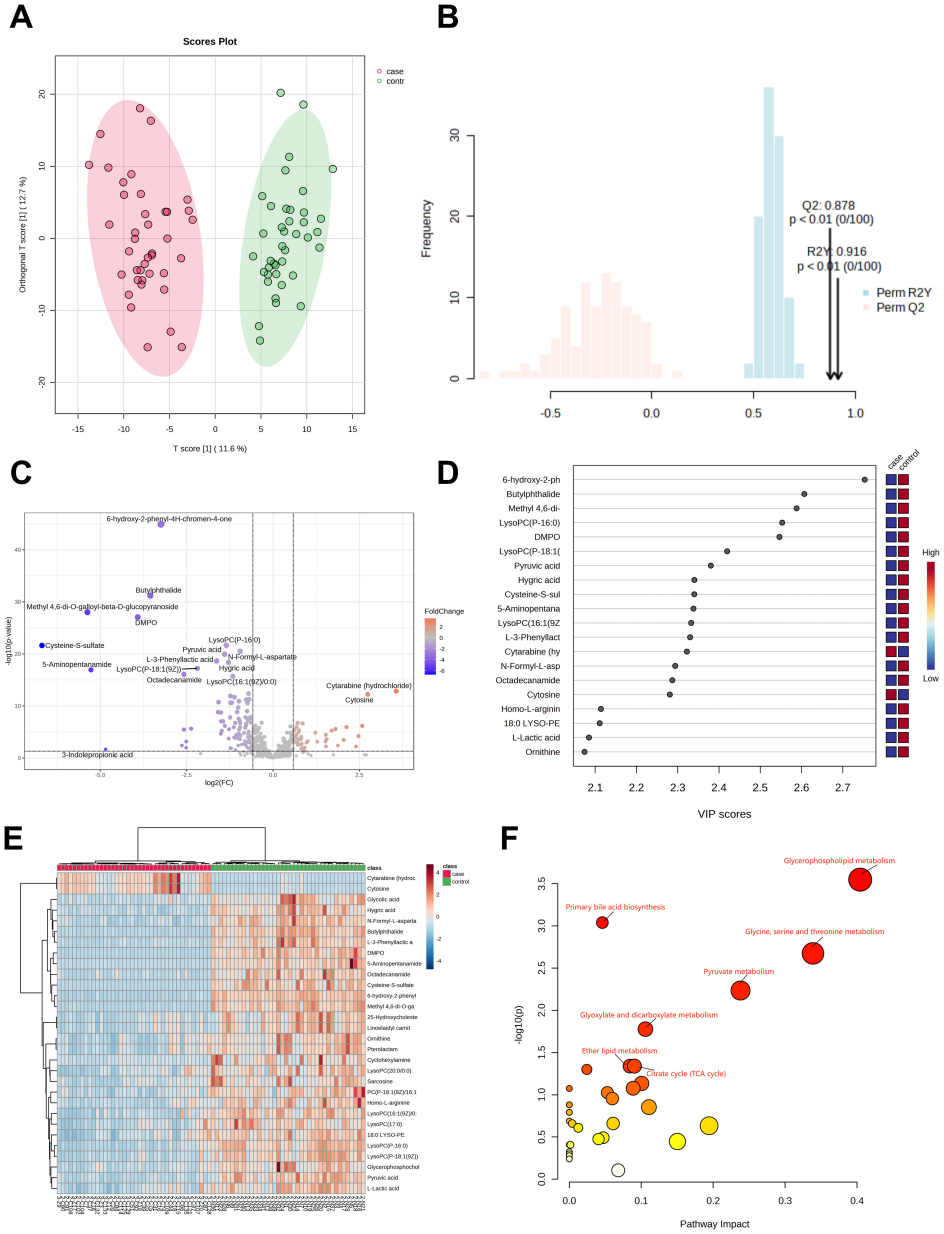
Metabolomic analysis of patients in the H3N2 group. OPLS-DA score diagram of pediatric H3N2 influenza patients and healthy controls **(A, B)**; Volcano plot of differential metabolites between children with H3N2 influenza and the healthy control group **(C)**; Top 20 metabolites with the highest VIP values **(D)**; Heatmap of the top 30 different metabolites between children with H3N2 influenza and healthy controls **(E)**; Enrichment analysis of the differentially abundant metabolite pathways in the H3N2 group vs. healthy controls **(F)**.

Plasma samples from 40 H3N2 patients and 40 healthy controls revealed 562 metabolites. OPLS-DA analysis (**Figures 2A, B**) showed clear group separation, with R²Y=0.916 and Q²=0.878 (*P*<0.01). A total of 79 differentially abundant metabolites were identified (VIP>1, *P*<0.05), including 26 upregulated and 53 downregulated metabolites (**Figure 2C**). Of these, 12 (15.1%) were glycerophospholipids, with 75% significantly downregulated in H3N2 cases.

The top 20 metabolites with the highest VIP values are shown in **Figure 2D**, while the top 30 differential metabolites are displayed in a heatmap (**Figure 2E**). KEGG pathway analysis indicated that the differentially abundant metabolites were primarily involved in glycerophospholipid metabolism, primary bile acid biosynthesis, glycine, serine, and threonine metabolism, pyruvate metabolism, glyoxylate and dicarboxylate metabolism, ether lipid metabolism, and the citrate cycle (**Figure 2F**; **Supplementary Table 2**).

Of the 12 glycerophospholipid metabolites with differences, 9 (75%) were significantly downregulated in H3N2 cases. These findings suggest that the metabolic disruptions observed in H3N2 are similar to those in H1N1, with predominant downregulation of glycerophospholipid pathways in both groups.

### 3.4 Comparative analysis of glycerophospholipid metabolism between H1N1 and H3N2

To further explore the metabolic differences between H1N1 and H3N2 influenza, a direct comparative analysis of metabolomic profiles was conducted between the two groups (47 H1N1 vs. 40 H3N2 cases). PCA and OPLS-DA analyses revealed that the overall metabolite distributions between H1N1 and H3N2 cases were highly similar, indicating that global metabolic changes induced by both viral strains share a common pattern. However, differential expression analysis identified one significantly different metabolite, Bergapten (Fold change=1.6668, p=0.024771), which was elevated in the H3N2 group relative to H1N1 cases (**Supplementary Figure 1**). Among the 562 detected metabolites, the concentration distribution was largely overlapping between the two groups, making it difficult to distinguish H1N1 from H3N2 based on metabolomic profiles alone. As shown in the volcano plot (**Supplementary Figure 1**), only a single metabolite reached the predefined differential threshold (Fold Change >1.5, p<0.05). Due to the small number of severe cases (10 in total), statistical power was insufficient to detect significant lipidomic differences between severe H1N1 and H3N2 cases. Future studies with a larger cohort of severe cases will be necessary to further explore the metabolic distinctions between these influenza subtypes and their implications for disease progression.

### 3.5 Association between glycerophospholipid metabolites and clinical indicators

**Figure 3:**
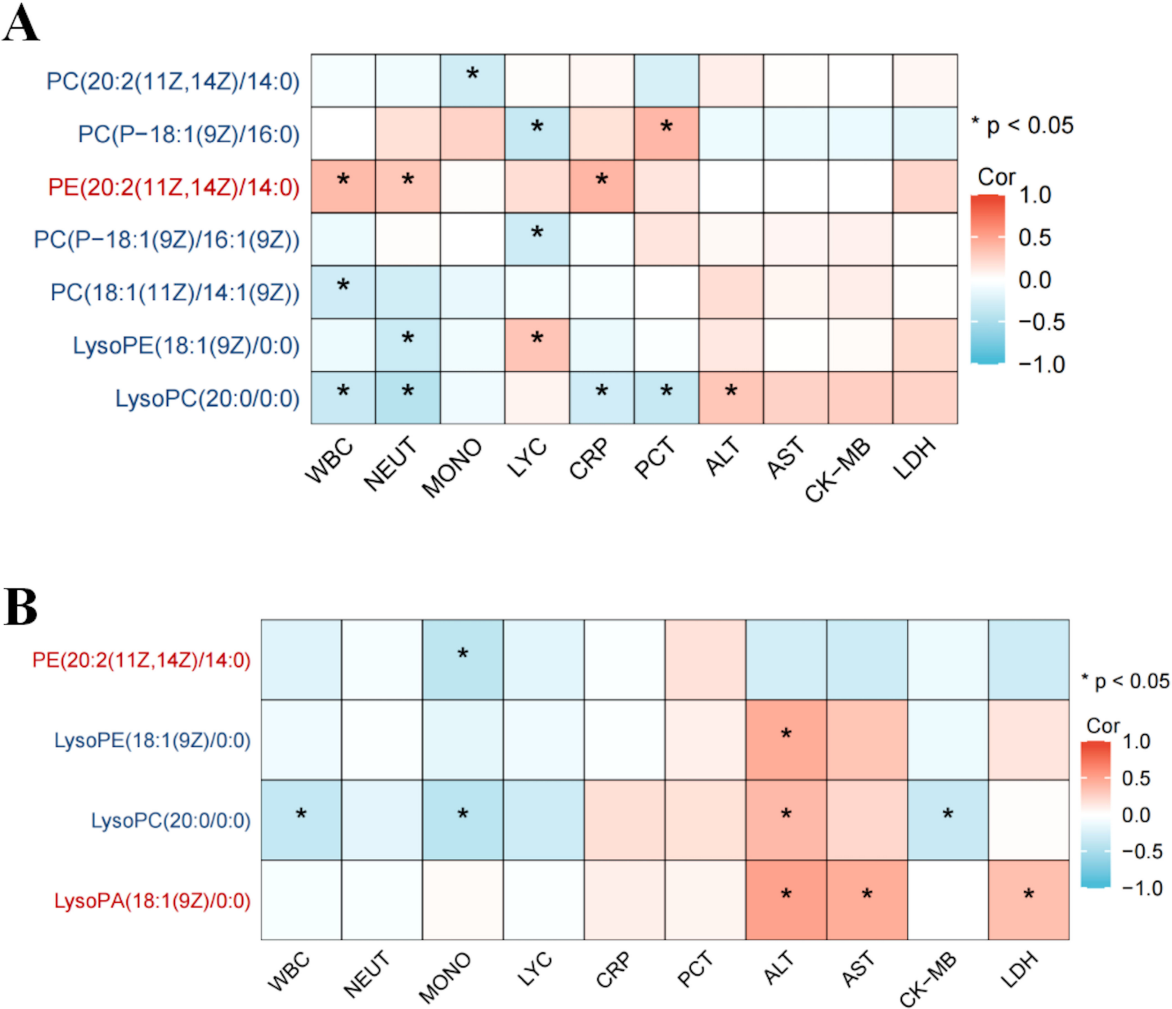
Association between differential glycerophospholipid metabolites and clinical indicators in children with influenza. Association between differential glycerophospholipid metabolites and clinical indicators in children the H1N1 patient and healthy control groups **(A)**; Association between differential glycerophospholipid metabolites and clinical indicators in children the H3N2 patient and healthy control groups **(B)**. * p < 0.05.

In H1N1 patients, 14 glycerophospholipid metabolites exhibited significant differences compared to healthy controls, with 11 (78.5%) being markedly downregulated. LysoPC(20:0/0.0), among the downregulated metabolites, negatively correlated with inflammatory markers (WBC, neutrophil count, CRP, and PCT), while the upregulated metabolite PC(P-18:1(9Z)/16:0) showed a positive correlation with validation markers (**Figure 3A**).

Similarly, in H3N2 patients, 12 glycerophospholipid metabolites differed significantly, with 9 (75%) being downregulated. LysoPC(20:0/0:0) showed a positive correlation with alanine aminotransferase (ALT) and a negative correlation with WBC count. Conversely, LysoPA(18:1(9Z)0:0), an upregulated metabolite, positively correlated with ALT, aspartate aminotransferase (AST), and lactate dehydrogenase (LDH) (**Figure 3B**).

### 3.6 Metabolomic analysis of severe and mild cases

**Figure 4:**
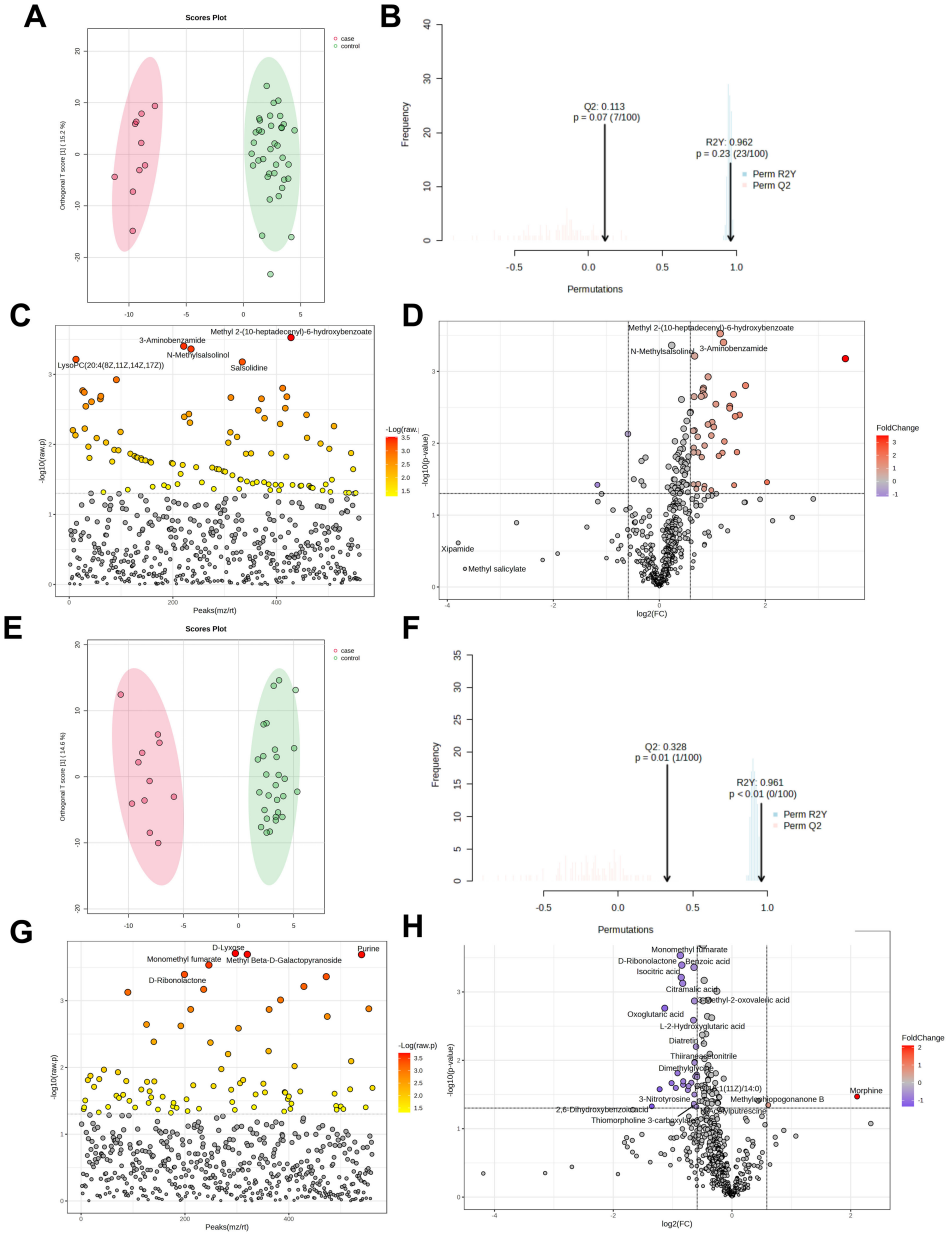
Metabolomic analysis of severe and mild groups of influenza patients. OPLS-DA Score Plot of Metabolites in Severe and Mild Cases of H1N1 Influenza infected Children **(A, B)**; Analysis of differential metabolites between H1N1 severe and mild pediatric patients (t-test) **(C)**; Volcano plot of differential metabolites between severe group and mild group in H1N1 patients **(D)**; OPLS-DA Score Plot of Metabolites in Severe and Mild Cases of H3N2 Influenza infected Children **(E, F)**; Analysis of differential metabolites between H3N2 severe and mild pediatric patients (t-test) **(G)**; Volcano plot of differential metabolites between severe group and mild group in H3N2 patients **(H)**.

The H1N1 cohort included 47 patients (10 severe, 37 mild). OPLS-DA score plots (**Figures 4A, B**) showed a cumulative R²Y of 0.962 and Q² of 0.113, indicating minimal distinction between severe and mild cases. Among 561 detected metabolites, 102 showed significant differences (*P*<0.05, **Figure 4C**), with 45 being further identified (43 upregulated, 2 downregulated, **Figure 4D**). Details of these differential metabolites are provided in **Supplementary Table 3**.

The H3N2 cohort comprised 40 patients (11 severe, 29 mild). OPLS-DA score plots (**Figures 4E, F**) yielded R²Y=0.961 and Q²=0.328, insufficient for clear differentiation between severity groups, potentially due to the small sample size. Of 561 metabolites, 83 differed significantly (*P*<0.05, **Figure 4G**), with 30 identified as significantly altered (2 upregulated, 28 downregulated, **Figure 4H**). Details of these differential metabolites are provided in **Supplementary Tables 3**.

Pathway enrichment analysis of the 45 H1N1 and 30 H3N2 differential metabolites indicated no significant pathway associations (*P*>0.05, **Supplementary Tables 4**, **5**). This suggests that glycerophospholipid metabolism disruption contributes to disease but lacks significant variation between severe and mild cases.

## 4 Discussion

The influenza virus spreads widely around the globe, resulting in seasonal epidemics. Influenza poses a significant threat not only to the elderly and individuals with underlying health conditions but also to children. Due to their immature immune systems, children are more susceptible to complications following influenza infection, posing a serious threat to human health. It is estimated that 3–5 million people are diagnosed with severe influenza annually, with complications and hospitalizations, and 250,000–500,000 deaths occur each year (Jané et al., 2019). During influenza seasons, influenza A virus is the most common virus, affecting both humans and animals. Influenza B, while highly contagious and causing small-scale local outbreaks, is less common during the epidemic season (Javanian et al., 2021). In the past 100 years, only three HA subtypes of influenza A viruses have caused pandemics in humans: H1N1, H2N2 and H3N2 (Webster and Govorkova, 2014). This indicates the significant challenge in controlling influenza, which causes substantial health and economic losses. Therefore, this research from the perspective of metabonomics is intended to explore the mechanism of influenza, providing the basis for early diagnosis and treatment.

In our study, clinical data from the H1N1, H3N2 and control groups revealed that the case groups had lower WBC and neutrophil counts compared with the control group, while the monocyte percentage was significantly higher. However, no significant differences were observed between the H1N1 and H3N2 groups in these immune cell counts. Changes in WBC count, neutrophil count and monocyte percentage suggest an inflammatory response. Numerous studies of the influenza A virus have shown that a decrease in peripheral blood leukocytes, lymphocytes and lymphocyte subpopulations is an immune process in the early stages of the disease (Ma et al., 2023). Neutrophils are the earliest immune cells to indicate lung infection and are an early significant feature of influenza virus infection (Gu et al., 2019). Regarding liver function clinical indicators, we found significant differences in ALT, AST, CK, CK-MB, and LDH levels between both the H1N1 and H3N2 groups and the control group (P<0.05). The elevated levels of ALT, AST, CK-MB, and LDH in the case groups indicate widespread tissue damage. These alterations are likely related to the immune response and the intensity of inflammation following viral infection (Ma et al., 2023). Notably, LDH levels were also significantly higher in both the H1N1 and H3N2 groups compared to controls. LDH levels in tissues are typically higher than in serum, and during inflammatory damage, LDH is released from tissues into the bloodstream, leading to increased serum levels (Hong et al., 2021). The significantly higher LDH levels in the H1N1 group compared to the H3N2 group suggest that H1N1 infection may cause more severe tissue damage.

The metabolomic results indicated significant differences in metabolites between the H1N1 and control groups, as well as between the H3N2 and control groups. These differences were primarily concentrated in the glycerophospholipid metabolism pathway, glycine, serine, and threonine metabolism pathways, pyruvate metabolism pathway, and primary bile acid biosynthesis pathway. Specifically, in the H1N1 group, 14 glycerophospholipid metabolites showed differences, with 11 significantly downregulated. In the H3N2 group, 12 glycerophospholipid metabolites showed differences, with 9 significantly downregulated. This indicates that glycerophospholipid metabolism is downregulated in both H1N1 and H3N2 influenza. Our results suggest a focus on the regulatory role of glycerophospholipid metabolites in the course of influenza infection, which could help in identifying accurate biomarkers for early detection, diagnosis and treatment.

Our results indicate a significant difference in glycerophospholipid metabolites between influenza patients and healthy controls, suggesting a potential role for these metabolites in the pathophysiology of influenza. However, no significant differences were observed in glycerophospholipid metabolites between mild and severe cases. The observed differences in glycerophospholipid metabolites between influenza patients and healthy controls highlight the impact of influenza on lipid metabolism. Glycerophospholipids are essential components of cell membranes and play crucial roles in cell signaling and membrane fluidity. Increased expression of sPLA2 and its activated form, sPLA2pS505, an enzyme that hydrolyzes sn-2 ester bonds of phospholipids, has been reported following influenza infection, causing metabolic damage to surfactants (Feng et al., 2023). The alteration in glycerophospholipid levels may reflect changes in membrane dynamics and immune cell function during influenza infection. Previous studies have shown that viral infections can disrupt lipid metabolism (Alketbi et al., 2021), which is consistent with our findings. Although we observed that most glycerophospholipid metabolites were downregulated in H1N1 and H3N2 influenza groups, there was no significant correlation between these metabolites and severity of influenza. This suggests that while glycerophospholipid metabolism is affected by the presence of the virus, the severity of the disease might not further influence these specific metabolic pathways. Additionally, metabolomic differences between viral causes of ARDS (e.g., COVID-19 and H1N1) have been reported, with significant alterations in glycerophospholipid metabolism. However, distinct differences were observed between bacterial pneumonia-associated ARDS and viral-associated ARDS in taurine and hypotaurine, arginine and proline, and histidine metabolism (Lee et al., 2024). These findings suggest that while viral infections, including influenza, can impact lipid metabolism, the specific metabolic responses may differ between viral and bacterial respiratory infections.

The metabolic pathways altered in H1N1 and H3N2 infections show considerable overlap, particularly in glycerophospholipid metabolism. However, whether these metabolic changes are influenza-specific or common across different respiratory infections remains an open question. Previous studies have reported that viral infections, including SARS-CoV-2 and respiratory syncytial virus (RSV), also induce significant changes in lipid metabolism, particularly in phospholipids, sphingolipids, and fatty acid metabolism (Lee et al., 2024). For instance, COVID-19 patients exhibit alterations in glycerophospholipid metabolism similar to those observed in our study (Lee et al., 2024). Additionally, bacterial pneumonia-associated metabolic changes appear to differ from those observed in viral infections, with major disruptions in taurine and hypotaurine metabolism, arginine and proline metabolism, and histidine metabolism (Lee et al., 2024). The similarity in metabolic alterations between H1N1 and H3N2 suggests that influenza viruses, regardless of subtype, may share common metabolic regulatory mechanisms. These changes may reflect a host metabolic adaptation to viral infection rather than a response specific to a particular influenza strain. Based on our findings and existing literature, we propose the following hypotheses: Glycerophospholipid metabolism downregulation is a general feature of influenza virus infection, rather than being subtype-specific. Both H1N1 and H3N2 groups exhibited significant downregulation in glycerophospholipid metabolites, suggesting that this pathway may be broadly affected by influenza viruses. However, the lack of significant metabolic differences between mild and severe cases in our study raises the possibility that while glycerophospholipid metabolism is altered during influenza infection, it may not be the primary determinant of disease severity.

Host small molecule metabolites play crucial roles in regulating the disease process. Metabolites are essential for driving vital cellular activities such as function and signal transduction. Among all tangible molecular substances (genes, transcripts, proteins and metabolites), metabolites have the closest relationship to the expressed phenotype because they are the final products of upstream biochemical processes (Rattray et al., 2018). From genotype (or genome) predictions of future events in the body to metabolotype (or metabolome) indications of ongoing small molecule changes, metabolomic analysis can help researchers better understand how viral infections affect host metabolism and immune responses (Wishart, 2019). Our study found that glycerophospholipid metabolism plays an essential regulatory role in children with influenza. While both H1N1 and H3N2 infections led to significant alterations in glycerophospholipid metabolism, we observed differences in the specific types and quantities of affected metabolites, suggesting virus-specific metabolic influences. LysoPA(18:1(9Z)/0:0) identified in the glycerophospholipid metabolism pathway screening, is a differential metabolite across all groups. This simple glycerophospholipid is a precursor of PA biosynthesis, significantly affecting various biochemical processes despite its low level in animal tissues (Aoki, 2004). LysoPA is involved in inflammatory lung diseases, acting as both a pro- and anti-inflammatory mediator (Wang et al., 2021). It is also considered a key product of glycolysis and glycerophospholipid metabolism, functioning as a mitogen and contributing to signal transduction through arachidonic acid provision (Hermansson et al., 2011). Changes in the lipid composition of the viral envelope can modulate viral fusion and thus correlate with infectivity. During viral infection, the organization and structure of the membrane is highly dependent on the lipid environment, suggesting that viral replication can be influenced by altering its structure (Ivanova et al., 2015). This suggests that differences in glycerophospholipids and their metabolites may regulate inflammation and immune response. Modulating these metabolites may inhibit influenza virus replication or enhance antiviral capacity.

We can draw the following conclusions regarding the changes in glycerophospholipid metabolites in influenza patients that most glycerophospholipid metabolites are significantly downregulated in the H1N1 patient group, suggesting that these metabolites may be consumed or their synthesis inhibited during influenza infection.

However, the H3N2 patient group, while also showing a similar downregulation trend, exhibited unique metabolic profiles. The specific types and quantities of altered metabolites differed, potentially due to the metabolic impacts specific to the H3N2 virus.

Specific glycerophospholipid metabolite LysoPC(20:0/0.0) showed a negative correlation with various inflammatory markers in H1N1 patients, indicating that it may play a crucial role in regulating the inflammatory response. PC(P-18:1(9Z)/16:0) was upregulated in H1N1 patients and positively correlated with validation markers, suggesting its possible association with the course or prognosis of influenza. An unnamed downregulated metabolite in H3N2 patients was positively correlated with ALT but negatively correlated with WBC count, implying its potential involvement in liver function regulation. LysoPA(18:1(9Z)0:0) was upregulated in H3N2 patients and positively correlated with ALT, AST and LDH, indicating its significant role in liver function and cellular damage. Phosphatidylcholine (PC), a predominant phospholipid in liver membranes, and its ratio when compared to phosphatidylethanolamine are implicated in triacylglycerol synthesis, potentially serving as a key regulator in the progression of liver injury. Sphingomyelin, another lipid identified, is associated with immune responses to infections. Changes in PC levels could affect phosphatidic acid metabolism, subsequently impacting the expression of PIK3CA and PIK3CG genes. This impacts the PI3K pathway and its associated upstream and downstream targets and regulates inflammation (Wu et al., 2024). Moreover, differences in lipidomic profiles between H1N1 and H3N2 suggest that while both subtypes affect similar pathways, the degree and nature of these metabolic alterations may depend on virus-specific factors such as viral protein-host interactions and immune evasion strategies (Rashid et al., 2023).

It has been shown that the parietal membrane of the host is enriched in sphingolipids and cholesterol, while glycerophospholipids and storage lipids are reduced after viral infection. Sphingolipids are important components of the RNA virus envelope and are involved in the inflammatory response during viral infection (Ma et al., 2022). This is in general agreement with our results. By analyzing the correlations between metabolites and disease markers, we can further understand the roles of these metabolites in the disease process. Given these virus-specific metabolic signatures, further comparative studies are warranted to determine whether these differences in glycerophospholipid metabolism contribute to variations in disease severity, immune response, or potential therapeutic targeting. The differential impact of various types of influenza virus infections on metabolites may provide clues for developing specific diagnostic markers and therapeutic targets in the future.

## 5 Conclusion

This study identified distinct metabolomic alterations in pediatric H1N1 and H3N2 influenza cases, particularly in glycerophospholipid metabolism. In H1N1 cases, most glycerophospholipid metabolites were downregulated and negatively correlated with inflammatory markers, while PC(P-18:1(9Z)/16:0) showed positive correlations with disease markers. In H3N2 cases, LysoPC(20:0/0:0) and LysoPA(18:1(9Z)/0:0) were associated with liver function markers (ALT, AST, LDH), suggesting potential hepatic involvement. These findings highlight the role of glycerophospholipid metabolism in influenza pathophysiology and its potential as a biomarker for disease monitoring and early diagnosis.

## Data Availability

The raw data supporting the conclusions of this article will be made available by the authors, without undue reservation.

## Supplementary material

Supplementary Figure 1 Differential Metabolite Analysis of H1N1 and H3N2 Children **(A)** Principal Component Analysis; **(B)** Orthogonal Partial Least Squares Discriminant Analysis; **(C)** Efficiency testing of OPLS-DA analysis; **(D)** Metabolic volcano map of H1N1 and H3N2 groups in children
