# Supplementary figures and images for: Plasma nontargeted metabolomics study of H1N1 and H3N2 influenza in children
Source: Front Cell Infect Microbiol. 2025 Apr 4;15:1537726. doi: 10.3389/fcimb.2025.1537726 (PMC12006178; doi:10.3389/fcimb.2025.1537726)

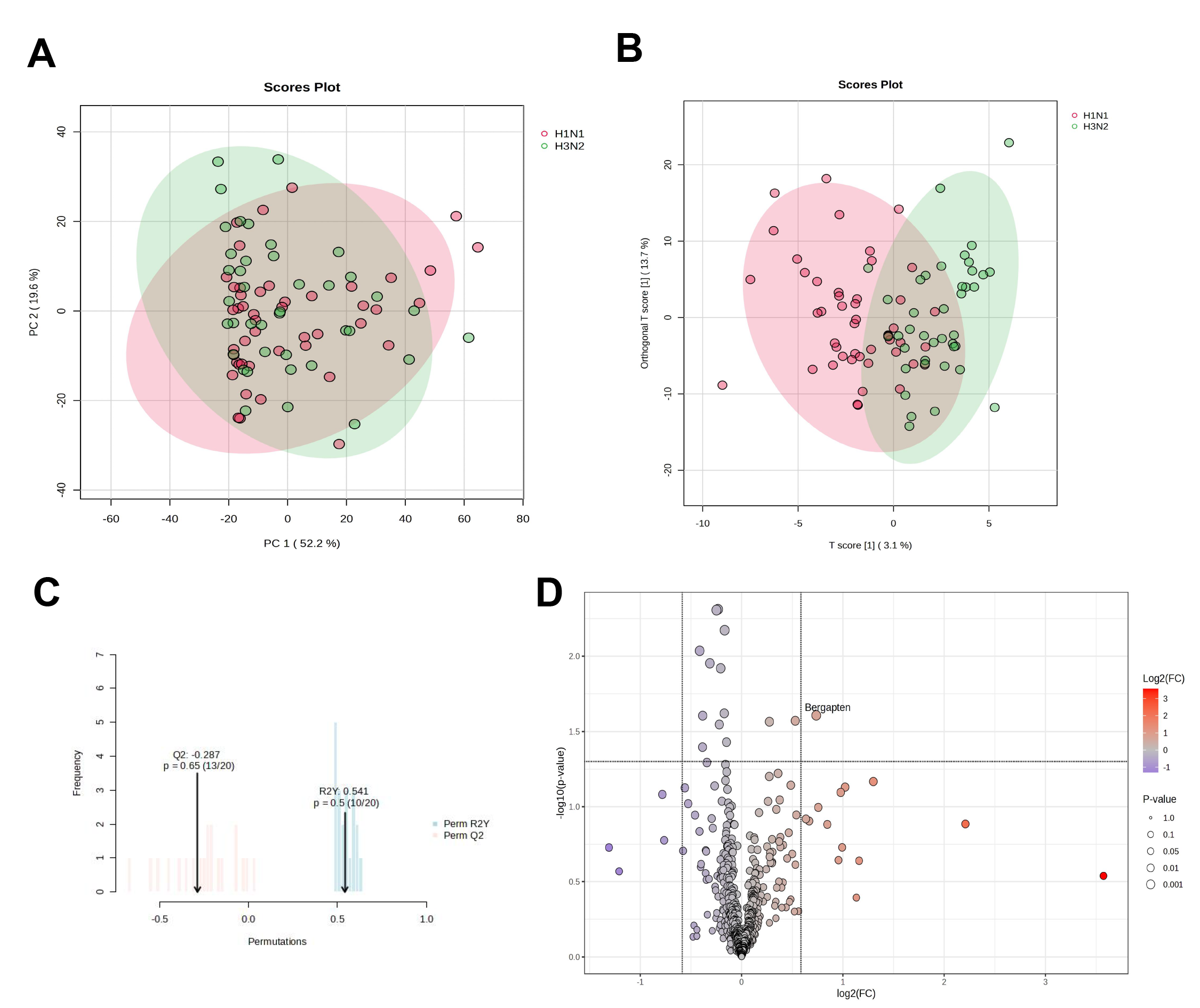

Supplement: Supplementary Figure 1 — Differential Metabolite Analysis of H1N1 and H3N2 Children (A) Principal Component Analysis; (B) Orthogonal Partial Least Squares Discriminant Analysis; (C) Efficiency testing of OPLS-DA analysis; (D) Metabolic volcano map of H1N1 and H3N2 groups in children [file Image1.tif]
