# Supplementary material for: Plasma nontargeted metabolomics study of H1N1 and H3N2 influenza in children
Source: Front Cell Infect Microbiol. 2025 Apr 4;15:1537726. doi: 10.3389/fcimb.2025.1537726 (PMC12006178; doi:10.3389/fcimb.2025.1537726)
Supplement: Supplementary file 2 [file Table1.docx]

**Supplementary Table 1 Comparison of the immune cell counts between the H1N1/H3N2 influenza and control groups**

| Groups | WBC count | Neutrophil count | Lymphocyte count | Monocyte count | Percentage of neutrophils | Percentage of lymphocytes | Percentage of monocytes |
| --- | --- | --- | --- | --- | --- | --- | --- |
| Control group | 6.38(5.81,6.98) | 2.91(2.40,3.97) | 2.92(2.19,3.40) | 0.39(0.34,0.52) | 46.83±10.87* | 44.35 (33.40,52.30 | 5.90(5.30,7.25) |
| HIN1 group | 5.24（3.77，6.79） | 1.86（1.22，3.36） | 2.56±1.38* | 0.42（0.34，0.65） | 42.11±21.09* | 47.57±20.58* | 8.3（7.2，11.7） |
| H3N2group | 5.56±2.59* | 2.1850(0.9925,3.2975) | 2.0700(1.5375,3.6800) | 0.52±0.23* | 40.58±19.74* | 47.11±19.59* | 9.7000(7.9250,13.2000) |
| P1 value | 0.003 | 0.002 | 0.074 | 0.325 | 0.185 | 0.371 | 0.000 |
| P2 value | 0.018 | 0.010 | 0.032 | 0.054 | 0.084 | 0.610 | 0.000 |
| P3 value | 0.936 | 0.997 | 0.612 | 0.627 | 0.729 | 0.916 | 0.178 |

*Normally distributed data. P1value, H1N1 patients compared with control patients; P2value, H3N2 patients compared with control patients; P3value, H1N1 patients compared with H3N2 patients.

**Supplementary Table 2 Enrichment analysis of differentially abundant metabolite pathways between the H1N1/H3N2 groups and healthy control group**

| Pathway name | Match Status | P | -log(p) | Holm P | FDR | Impact |
| --- | --- | --- | --- | --- | --- | --- |
| H1N1 vs. Healthy Control |  |  |  |  |  |  |
| [Glycine, serine and threonine metabolism](https://www.metaboanalyst.ca/MetaboAnalyst/Secure/pathway/PathResultView.xhtml) | [5/33](https://www.metaboanalyst.ca/MetaboAnalyst/Secure/pathway/PathResultView.xhtml) | 4.9632×10^4 | 3.3042 | 0.041691 | 0.03163 | 0.3387 |
| [**Glycerophospholipid metabolism**](https://www.metaboanalyst.ca/MetaboAnalyst/Secure/pathway/PathResultView.xhtml) | [**5/36**](https://www.metaboanalyst.ca/MetaboAnalyst/Secure/pathway/PathResultView.xhtml) | 7.531×10^4 | 3.1231 | 0.062507 | 0.03163 | 0.40432 |
| [Pyruvate metabolism](https://www.metaboanalyst.ca/MetaboAnalyst/Secure/pathway/PathResultView.xhtml) | [3/22](https://www.metaboanalyst.ca/MetaboAnalyst/Secure/pathway/PathResultView.xhtml) | 0.010281 | 1.988 | 0.84306 | 0.28787 | 0.23794 |
| [Primary bile acid biosynthesis](https://www.metaboanalyst.ca/MetaboAnalyst/Secure/pathway/PathResultView.xhtml) | [4/46](https://www.metaboanalyst.ca/MetaboAnalyst/Secure/pathway/PathResultView.xhtml) | 0.014798 | 1.8298 | 1.0 | 0.31076 | 0.03619 |
| [Glyoxylate and dicarboxylate metabolism](https://www.metaboanalyst.ca/MetaboAnalyst/Secure/pathway/PathResultView.xhtml) | [3/32](https://www.metaboanalyst.ca/MetaboAnalyst/Secure/pathway/PathResultView.xhtml) | 0.028653 | 1.5428 | 1.0 | 0.4092 | 0.10582 |
| [Cysteine and methionine metabolism](https://www.metaboanalyst.ca/MetaboAnalyst/Secure/pathway/PathResultView.xhtml) | [3/33](https://www.metaboanalyst.ca/MetaboAnalyst/Secure/pathway/PathResultView.xhtml) | 0.031067 | 1.5077 | 1.0 | 0.4092 | 0.05983 |
| [Arginine biosynthesis](https://www.metaboanalyst.ca/MetaboAnalyst/Secure/pathway/PathResultView.xhtml) | [2/14](https://www.metaboanalyst.ca/MetaboAnalyst/Secure/pathway/PathResultView.xhtml) | 0.0341 | 1.4672 | 1.0 | 0.4092 | 0.26396 |
| H3N2 vs. Healthy Control |  |  |  |  |  |  |
| [Glycerophospholipid metabolism](https://www.metaboanalyst.ca/MetaboAnalyst/Secure/pathway/PathResultView.xhtml) | [5/36](https://www.metaboanalyst.ca/MetaboAnalyst/Secure/pathway/PathResultView.xhtml) | 2.8347×10^4 | 3.5475 | 0.023811 | 0.023811 | 0.40432 |
| [Primary bile acid biosynthesis](https://www.metaboanalyst.ca/MetaboAnalyst/Secure/pathway/PathResultView.xhtml) | [5/46](https://www.metaboanalyst.ca/MetaboAnalyst/Secure/pathway/PathResultView.xhtml) | 9.1398×10^4 | 3.0391 | 0.07586 | 0.038387 | 0.04596 |
| [Glycine, serine and threonine metabolism](https://www.metaboanalyst.ca/MetaboAnalyst/Secure/pathway/PathResultView.xhtml) | [4/33](https://www.metaboanalyst.ca/MetaboAnalyst/Secure/pathway/PathResultView.xhtml) | 0.0021181 | 2.6741 | 0.17369 | 0.059307 | 0.3387 |
| [Pyruvate metabolism](https://www.metaboanalyst.ca/MetaboAnalyst/Secure/pathway/PathResultView.xhtml) | [3/22](https://www.metaboanalyst.ca/MetaboAnalyst/Secure/pathway/PathResultView.xhtml) | 0.0058254 | 2.2347 | 0.47185 | 0.12233 | 0.23794 |
| [Glyoxylate and dicarboxylate metabolism](https://www.metaboanalyst.ca/MetaboAnalyst/Secure/pathway/PathResultView.xhtml) | [3/32](https://www.metaboanalyst.ca/MetaboAnalyst/Secure/pathway/PathResultView.xhtml) | 0.016704 | 1.7772 | 1.0 | 0.28063 | 0.10582 |
| [Ether lipid metabolism](https://www.metaboanalyst.ca/MetaboAnalyst/Secure/pathway/PathResultView.xhtml) | [2/20](https://www.metaboanalyst.ca/MetaboAnalyst/Secure/pathway/PathResultView.xhtml) | 0.045787 | 1.3393 | 1.0 | 0.52574 | 0.08434 |
| [Citrate cycle (TCA cycle)](https://www.metaboanalyst.ca/MetaboAnalyst/Secure/pathway/PathResultView.xhtml) | [2/20](https://www.metaboanalyst.ca/MetaboAnalyst/Secure/pathway/PathResultView.xhtml) | 0.045787 | 1.3393 | 1.0 | 0.52574 | 0.09046 |

**Supplementary Table 3 Differential metabolites between severe and mild cases of H1N1/H3N2 influenza in children**

| Name | FC | log2(FC) | raw.pval | -log10(P) |
| --- | --- | --- | --- | --- |
| **H1N1 severe vs mild** |  |  |  |  |
| Methyl 2-(10-heptadecenyl)-6-hydroxybenzoate | 2.2166 | 1.1483 | 0.00029776 | 3.5261 |
| 3-Aminobenzamide | 2.3142 | 1.2105 | 0.00039562 | 3.4027 |
| LysoPC(20:4(8Z,11Z,14Z,17Z)) | 1.5867 | 0.66604 | 0.00061028 | 3.2145 |
| Salsolidine | 11.398 | 3.5107 | 0.00066483 | 3.1773 |
| Hydroxypyruvic acid | 1.8921 | 0.91998 | 0.0011925 | 2.9236 |
| Hexadecanedioic acid mono-L-carnitine ester | 3.0876 | 1.6265 | 0.0015759 | 2.8025 |
| Estrone | 1.7803 | 0.83216 | 0.0017091 | 2.7672 |
| LysoPC(22:6(4Z,7Z,10Z,13Z,16Z,19Z)) | 1.7706 | 0.82425 | 0.0018066 | 2.7431 |
| Decanoylcarnitine | 1.7689 | 0.82281 | 0.0020516 | 2.6879 |
| 3-Methylglutarylcarnitine | 2.6388 | 1.3999 | 0.002086 | 2.6807 |
| L-Octanoylcarnitine | 1.7987 | 0.84694 | 0.0021433 | 2.6689 |
| 3-Hydroxyvaleric acid | 1.7297 | 0.79054 | 0.0022317 | 2.6514 |
| N4-Acetylcytidine | 1.5755 | 0.65584 | 0.0022465 | 2.6485 |
| LysoPA(16:0/0:0) | 2.0946 | 1.0667 | 0.002858 | 2.5439 |
| Dihydro-2-methoxy-2-methyl-3(2H)-thiophenone | 2.5112 | 1.3284 | 0.0030426 | 2.5168 |
| S-Propyl-L-cysteine | 2.5087 | 1.3269 | 0.0032563 | 2.4873 |
| (6beta,22E)-6-Hydroxystigmasta-4,22-dien-3-one | 1.5041 | 0.58894 | 0.0036826 | 2.4338 |
| 2-Hydroxyhexanoic acid | 2.8556 | 1.5138 | 0.0040394 | 2.3937 |
| Calcitriol | 2.695 | 1.4303 | 0.0042277 | 2.3739 |
| 2-Methyltetrahydrofuran-3-one | 1.9957 | 0.9969 | 0.0050965 | 2.2927 |
| LysoPA(18:1(9Z)/0:0) | 1.8879 | 0.91682 | 0.005483 | 2.261 |
| Dodecanoylcarnitine | 2.0384 | 1.0274 | 0.0059469 | 2.2257 |
| Malonic acid | 1.5825 | 0.66219 | 0.0066359 | 2.1781 |
| Progesterone | 1.558 | 0.63965 | 0.0067214 | 2.1725 |
| PC(P-18:1(11Z)/22:2(13Z,16Z)) | 0.66315 | -0.59259 | 0.0074109 | 2.1301 |
| trans-Hexadec-2-enoyl carnitine | 1.9742 | 0.98126 | 0.0078115 | 2.1073 |
| Prolylhydroxyproline | 1.6511 | 0.72345 | 0.0081255 | 2.0901 |
| 1-Phenyl-1,3-heptadecanedione | 2.341 | 1.2272 | 0.0094483 | 2.0246 |
| (R)-Leucic acid | 1.8075 | 0.854 | 0.01078 | 1.9674 |
| GSK0660 | 1.5667 | 0.64772 | 0.011553 | 1.9373 |
| Arecaidine | 2.7602 | 1.4648 | 0.0133 | 1.8762 |
| Glycolic acid | 1.6608 | 0.73191 | 0.013438 | 1.8717 |
| N-trans-Cinnamoylglycine | 1.5655 | 0.64664 | 0.013446 | 1.8714 |
| cis-5-Tetradecenoylcarnitine | 2.2821 | 1.1904 | 0.0136 | 1.8665 |
| Deoxyelephantopin | 2.1763 | 1.1219 | 0.014989 | 1.8242 |
| 2-Hydroxy-3-methylbutyric acid | 1.7419 | 0.80063 | 0.015618 | 1.8064 |
| 3α,4,7,7α-Tetrahydro-4-hydroxy-1H-isoindole-1,3(2H)-dione | 4.0879 | 2.0313 | 0.034851 | 1.4578 |
| L-Theanine | 1.6628 | 0.73359 | 0.036893 | 1.4331 |
| 3-Methoxy-4-hydroxyphenylethyleneglycol sulfate | 1.5623 | 0.64363 | 0.037255 | 1.4288 |
| Chenodeoxycholic acid glycine conjugate | 0.44483 | -1.1687 | 0.037894 | 1.4214 |
| 2-Hydroxyoctanoic acid | 2.6593 | 1.4111 | 0.038311 | 1.4167 |
| (R)-3-Hydroxybutyric acid | 1.8051 | 0.85208 | 0.039197 | 1.4067 |
| Hydroxyprolyl-asparagine | 1.7946 | 0.84369 | 0.043203 | 1.3645 |
| N-Acetylvaline | 1.6234 | 0.69904 | 0.043207 | 1.3644 |
| 4-Isopropylbenzoic acid | 1.9727 | 0.98017 | 0.044892 | 1.3478 |
| **H3N2 severe vs mild** |  |  |  |  |
| Monomethyl fumarate | 0.54713 | -0.87005 | 0.00029236 | 3.5341 |
| D-Ribonolactone | 0.55655 | -0.84542 | 0.0004049 | 3.3927 |
| Benzoic acid | 0.64213 | -0.63906 | 0.00043682 | 3.3597 |
| Isocitric acid | 0.55282 | -0.85511 | 0.00061264 | 3.2128 |
| Citramalic acid | 0.56251 | -0.83006 | 0.00074889 | 3.1256 |
| 3-Methyl-2-oxovaleric acid | 0.64513 | -0.63233 | 0.0013546 | 2.8682 |
| Oxoglutaric acid | 0.45616 | -1.1324 | 0.0017267 | 2.7628 |
| L-2-Hydroxyglutaric acid | 0.63785 | -0.64872 | 0.0025918 | 2.5864 |
| Diatretin 2 | 0.65731 | -0.60535 | 0.0063008 | 2.2006 |
| Thiiraneacetonitrile | 0.64626 | -0.62982 | 0.010578 | 1.9756 |
| Dimethylglycine | 0.64555 | -0.63141 | 0.010809 | 1.9662 |
| L-Octanoylcarnitine | 0.52957 | -0.91709 | 0.015424 | 1.8118 |
| D-Pantothenic Acid | 0.66199 | -0.59513 | 0.016187 | 1.7908 |
| 4-Acetamidobutyric-Acid | 0.6519 | -0.61728 | 0.017234 | 1.7636 |
| Glycolic acid | 0.66552 | -0.58744 | 0.017503 | 1.7569 |
| O-propanoyl-carnitine | 0.5661 | -0.82087 | 0.020264 | 1.6933 |
| Hypogeic acid | 0.62242 | -0.68404 | 0.021266 | 1.6723 |
| LysoPA(18:1(9Z)/0:0) | 0.49392 | -1.0176 | 0.021534 | 1.6669 |
| Prolylhydroxyproline | 0.56535 | -0.82279 | 0.022769 | 1.6427 |
| Aesculetin | 0.60238 | -0.73125 | 0.02382 | 1.6231 |
| Glycine | 0.51916 | -0.94575 | 0.025346 | 1.5961 |
| Acetylglycine | 0.42963 | -1.2188 | 0.026483 | 1.577 |
| Glutaminylaspartic acid | 0.59728 | -0.74353 | 0.027182 | 1.5657 |
| PC(18:1(11Z)/14:0) | 0.64442 | -0.63392 | 0.031611 | 1.5002 |
| Morphine | 4.3108 | 2.108 | 0.033859 | 1.4703 |
| 3-Nitrotyrosine | 0.64082 | -0.64201 | 0.043013 | 1.3664 |
| Methylophiopogonanone B | 1.5268 | 0.61047 | 0.045043 | 1.3464 |
| Thiomorpholine 3-carboxylate | 0.64079 | -0.64209 | 0.045589 | 1.3411 |
| 2,6-Dihydroxybenzoic acid | 0.3921 | -1.3507 | 0.047084 | 1.3271 |
| N-Acetylputrescine | 0.66046 | -0.59845 | 0.047852 | 1.3201 |

**Supplementary Table 4 Pathway analysis of differential metabolites between severe and mild cases of H1N1 influenza in children**

|  | Total | Expected | Hits | Raw p | Log10(p) | Holm adjust | FDR | Impact |
| --- | --- | --- | --- | --- | --- | --- | --- | --- |
| Steroid hormone biosynthesis | 87 | 0.4419 | 2 | 0.067971 | 1.1677 | 1 | 1 | 0.10302 |
| Butanoate metabolism | 15 | 0.07619 | 1 | 0.073857 | 1.1316 | 1 | 1 | 0 |
| Glyoxylate and dicarboxylate metabolism | 32 | 0.16254 | 1 | 0.15175 | 0.81886 | 1 | 1 | 0.21958 |
| Glycine, serine and threonine metabolism | 33 | 0.16762 | 1 | 0.15615 | 0.80646 | 1 | 1 | 0.04653 |
| Fatty acid degradation | 39 | 0.1981 | 1 | 0.18212 | 0.73964 | 1 | 1 | 0 |
| Steroid biosynthesis | 41 | 0.20825 | 1 | 0.19062 | 0.71983 | 1 | 1 | 0.00208 |
| Primary bile acid biosynthesis | 46 | 0.23365 | 1 | 0.21154 | 0.67462 | 1 | 1 | 0.00977 |
| Fatty acid biosynthesis | 47 | 0.23873 | 1 | 0.21566 | 0.66623 | 1 | 1 | 0 |

**Supplementary Table 5 Pathway analysis of differential metabolites between severe and mild cases of H3N2 influenza in children**

|  | Total | Expected | Hits | Raw p | -Log10(p) | Holm adjust | FDR | Impact |
| --- | --- | --- | --- | --- | --- | --- | --- | --- |
| Citrate cycle (TCA cycle) | 20 | 0.11429 | 2 | 0.0052306 | 2.2814 | 0.41845 | 0.27967 | 0.10354 |
| Lipoic acid metabolism | 28 | 0.16 | 2 | 0.010161 | 1.9931 | 0.80274 | 0.27967 | 0.0017 |
| Glyoxylate and dicarboxylate metabolism | 32 | 0.18286 | 2 | 0.013175 | 1.8802 | 1 | 0.27967 | 0.10582 |
| Glycine, serine and threonine metabolism | 33 | 0.18857 | 2 | 0.013984 | 1.8544 | 1 | 0.27967 | 0.33407 |
| Valine, leucine and isoleucine biosynthesis | 8 | 0.045714 | 1 | 0.044908 | 1.3477 | 1 | 0.71853 | 0 |
| Arginine biosynthesis | 14 | 0.08 | 1 | 0.077404 | 1.1112 | 1 | 0.9454 | 0 |
| Butanoate metabolism | 15 | 0.085714 | 1 | 0.082723 | 1.0824 | 1 | 0.9454 | 0 |
| Pantothenate and CoA biosynthesis | 20 | 0.11429 | 1 | 0.10891 | 0.96292 | 1 | 1 | 0.0068 |
| Alanine, aspartate and glutamate metabolism | 28 | 0.16 | 1 | 0.14944 | 0.82555 | 1 | 1 | 0.04808 |
| Glutathione metabolism | 28 | 0.16 | 1 | 0.14944 | 0.82555 | 1 | 1 | 0.08873 |
| Porphyrin metabolism | 31 | 0.17714 | 1 | 0.1642 | 0.78462 | 1 | 1 | 0 |
| Arginine and proline metabolism | 36 | 0.20571 | 1 | 0.18831 | 0.72512 | 1 | 1 | 0.04651 |
| Fatty acid degradation | 39 | 0.22286 | 1 | 0.20248 | 0.69362 | 1 | 1 | 0 |
| Valine, leucine and isoleucine degradation | 40 | 0.22857 | 1 | 0.20715 | 0.68371 | 1 | 1 | 0.01084 |
| Primary bile acid biosynthesis | 46 | 0.26286 | 1 | 0.23468 | 0.62952 | 1 | 1 | 0.00758 |
| Drug metabolism - cytochrome P450 | 55 | 0.31429 | 1 | 0.27438 | 0.56164 | 1 | 1 | 0.10145 |
